# Correction: Spatial Distribution of Fungal Communities in an Arable Soil

**DOI:** 10.1371/journal.pone.0154290

**Published:** 2016-04-21

**Authors:** The PLOS ONE Staff

## Notice of Republication

This article was republished on April 13, 2016, to correct errors in the figures in the PDF that were introduced during the typesetting process. The publisher apologizes for the errors. Please download this article again to view the correct version. The originally published, uncorrected article and the republished, corrected article are provided here for reference.

## Supporting Information

S1 File Originally published, uncorrected article. (PDF)

S2 File Republished, corrected article. (PDF)

## References

[1] Moll J, Hoppe B, König S, Wubet T, Buscot F, Krüger D (2016) Spatial Distribution of Fungal Communities in an Arable Soil. PLoS ONE 11(2): e0148130 doi:10.1371/journal.pone.0148130 26840453 10.1371/journal.pone.0148130 PMC4740416

