# Supplementary figures and images for: Spatial Distribution of Fungal Communities in an Arable Soil
Source: PLoS One. 2016 Feb 3;11(2):e0148130. doi: 10.1371/journal.pone.0148130 (PMC4740416; doi:10.1371/journal.pone.0148130)

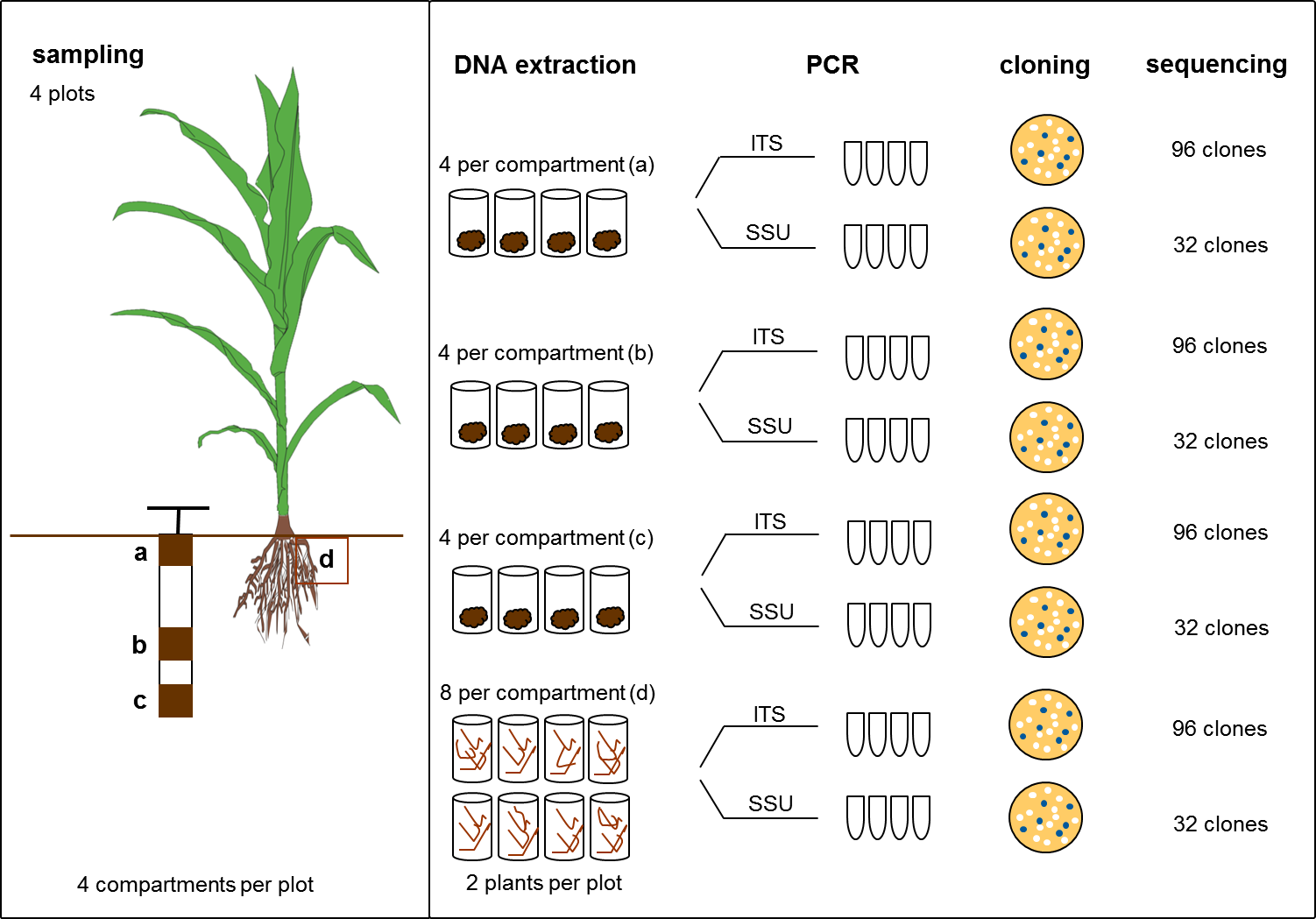

Supplement: S1 Fig — Sampling took place in summer, autumn and winter over two annual cycles (2009 and 2010/2011). Four replicated plots were sampled. Within one plot four compartments a) 0–10 cm, b) 40–50 cm and c) 60–70 cm soil depth and d) maize roots were analyzed. Within one plot each of three soil compartments a-c consisted of soil from ten soil cores of respective depth, so that finally four replicated samples from four plots represented each soil compartment. For sampling the root compartment d, two maize plants per plot were grubbed and approximately 20 fine roots were randomly taken. DNA extraction of all sampled compartments was performed separately for each plot. Genomic DNA was used 1) for the amplification of the ITS/LSU region and 2) for the amplification of the SSU region. Purified amplicons of the biological replicates were pooled and used for one cloning reaction for each compartment. Finally, 96 clones for the ITS/LSU region and 32 clones for the SSU region were screened by Sanger sequencing. (TIF) [file pone.0148130.s001.tif]

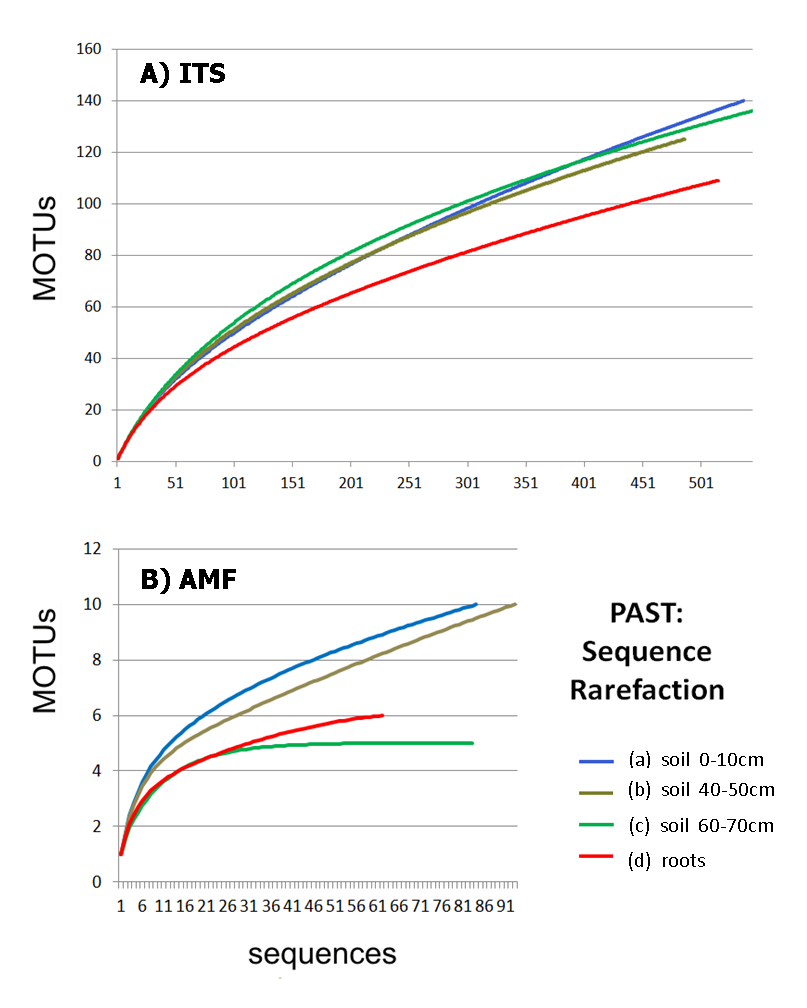

Supplement: S2 Fig — (TIF) [file pone.0148130.s002.tif]

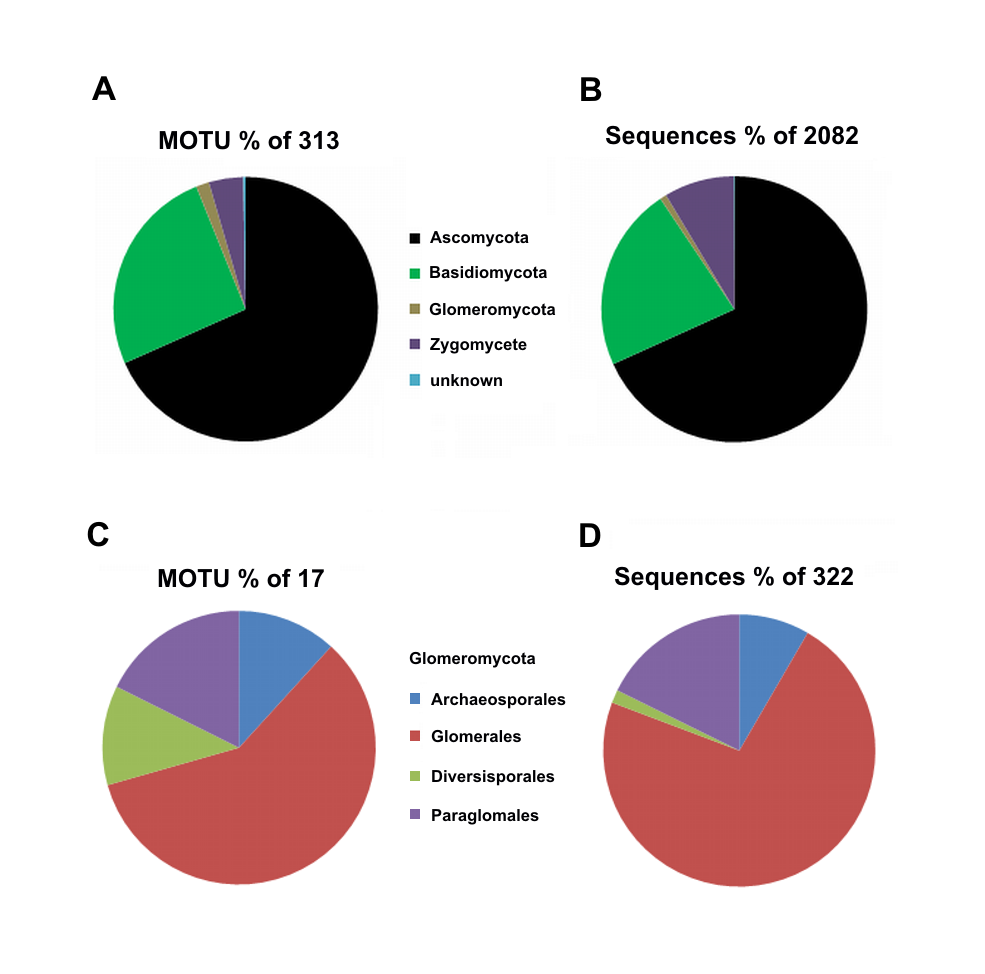

Supplement: S3 Fig — A) Distribution among MOTUs for ITS dataset by phylum, B) distribution among sequences for ITS dataset by phylum, C) distribution among MOTUs for AMF dataset by order and D) distribution among sequences for AMF dataset by order. (TIF) [file pone.0148130.s003.tif]

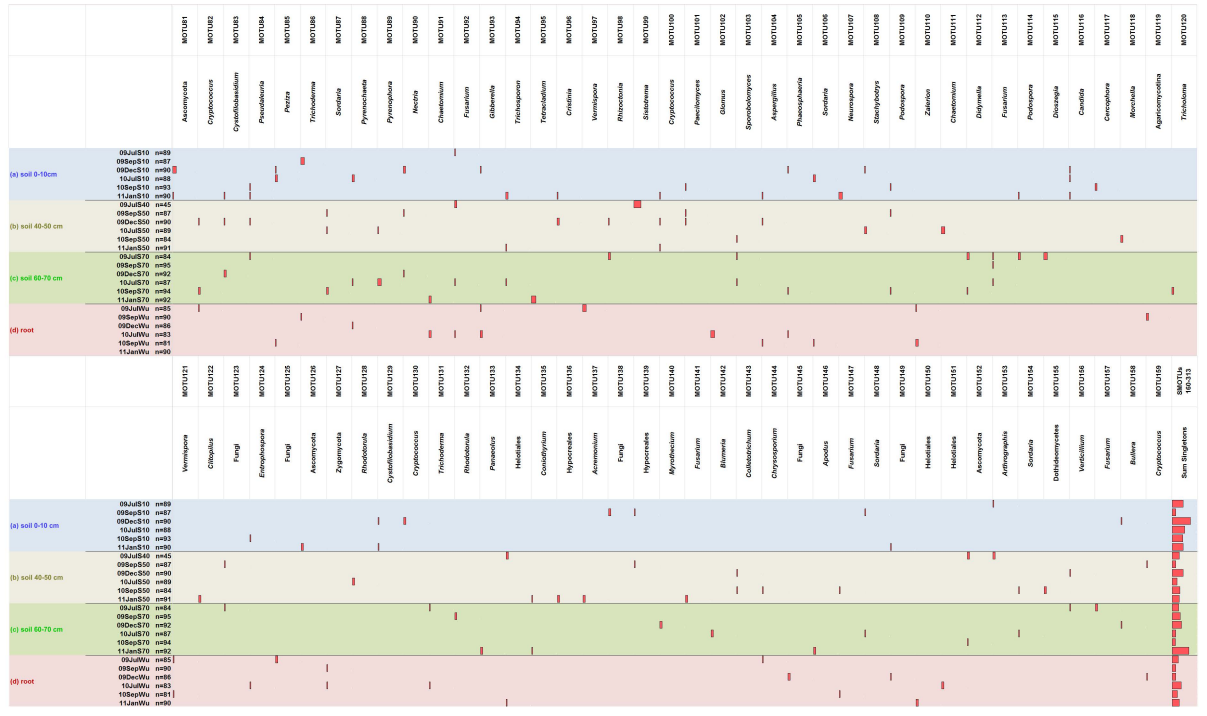

Supplement: S4 Fig — Red bars represent relative abundance of respective MOTU in the sample, smallest bars are 1 sequence. The sample names to the left contain the year (09 = 2009, 10 = 2010, 11 = 2011), abbreviated month and depth, number of sequences. Sample names contain the year (09 = 2009, 10 = 2010, 11 = 2011), abbreviated month (Jul = July, Sep = September, Dec = December, Jan = January) and depth (S10 = Soil 0–10 cm, S50 = Soil 40–50 cm, S70 = Soil 60–70 cm), n = number of quality checked sequences. (PDF) [file pone.0148130.s004.pdf]
