# Supplementary material for: Spatial Distribution of Fungal Communities in an Arable Soil
Source: PLoS One. 2016 Feb 3;11(2):e0148130. doi: 10.1371/journal.pone.0148130 (PMC4740416; doi:10.1371/journal.pone.0148130)
Supplement: S1 File — (PDF) [file pone.0148130.s005.pdf]

Spatial distribution of fungal communities in an arable soil

Manuscript for PLoS ONE
Julia Moll, Björn Hoppe, Stephan König, Tesfaye Wubet, François Buscot and Dirk Krüger

Corresponding author:
Julia Moll, e-mail: julia.moll@ufz.de, phone: +49 345 5585225, fax: +49 345 5585329
UFZ – Helmholtz Centre for Environmental Research, Department of Soil Ecology, Theodor-Lieser-Str. 4, 06120 Halle (Saale) , Germany

Supplemental information – File S1

Fungal community structure analysis by denaturing gradient gel electrophoresis (DGGE) fingerprints

Introduction

A preliminary, but detailed fingerprint analysis was performed to investigate the distribution of fungal communities between individual maize plots (biological replicates). The aim was to prove whether pooling of biological replicates to one cloning reaction allows drawing reliable conclusion on fungal key players across compartments.

DGGE method

Approximately 250 bp of the variable D1 region of fungal LSU of all samples were amplified with the primer set NL1fGC and LS2r (Cocolin et al. 2000). PCR was performed in 50 µl-reaction mixture containing 10 µl FIREPol 5x Master Mix (Solis BioDyne, Tartu, Estonia), 25 µM of each Primer and approximately 20 ng template DNA. PCR was performed with an initial denaturation step at 95°C for 5 min followed by 35 cycles at 95°C for 40 s, 55°C for 30 s and 72°C for 1 min. Extension was completed with a final step of 72°C for 10 min. Approximately 90 ng of PCR product was run per lane on an 8 % polyacrylamide gel with 1x TAE buffer. The DGGE gels with 25-55 % denaturant gradient (100 % denaturing is defined as containing 7 M urea and 40 % formamide) were run in an INGENY phorU gel system (Ingeny International, Goes, Netherlands) at 160 V for 6 h. Gels were stained in 1x GelRed (VWR, Darmstadt, Germany) for 30 min, then analysed under UV light and digitally captured using the Biovision gel documentation system (Vilber Lourmat, Eberhardzell, Germany).

DGGE results

Preliminary fungal community structure screening for samplings in 2009 revealed homogenous distribution of fungal phylotypes among biological replicates (maize plots). DGGE gels showed particular bands for the respective soil compartments (Figs A and B). For instance, bands 3, 4, 8 and 9 were observed in all biological replicates for respective compartments and sampling dates (Fig A). Bands 1 and 2 were commonly apparent in bulk

soil compartments a and b but became less dominant in 60-70 cm soil depth (compartment c) especially in September and December 2009. 09SepS70 samples stand out with three highly dominant phylotypes (bands 5-7). This mirrors notable Sanger sequencing results for compartment c in September 2009 (Fig 1, S4 Fig). About 84 % of sequences belonged to only the three genera *Rhodotorula*, *Cryptococcus* and *Davidiella*.
The same homogenous pattern among biological replicates was observed for compartment d (Fig B). Band 1 was observed for July in all collected root samples, while band 2 was found in all roots in December 2009.

In summary, these results support the chosen experimental design of pooling biological replicates to assess most dominant fungal key species for the different compartments. The validity of results is additionally supported by our recently published comprehensive study which demonstrated by F-ARISA fingerprints (fungal automated ribosomal intergenic spacer analysis) that differences between vertical soil horizons were higher than within-group differences of biological replicates (Moll et al. 2015).

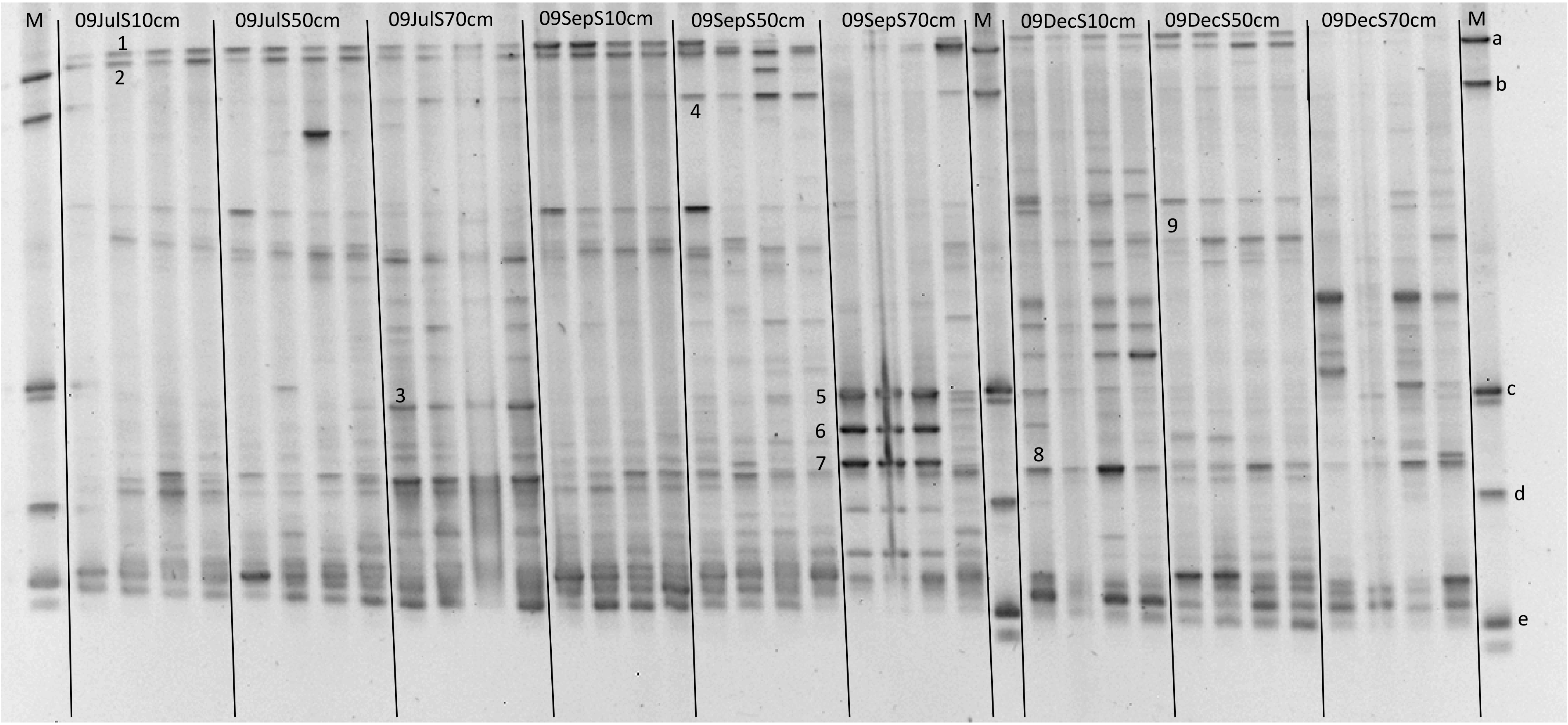

Fig A. DGGE results for fungal community structure across the three bulk soil compartments a, b and c for July, September and December 2009. Sample names contain the year (09 = 2009), abbreviated month (Jul = July, Sep = September, Dec = December) and depth (S10 = Soil 0-10 cm, S50 = Soil 40-50 cm, S70 = Soil 60-70 cm). M = marker (a) *Mortierella cf. verticiellata, b) Mortierella cf. alpina, c) Rhodotorula sp., d) Exophiala sp., e) Verticillium cf. nigrescens*). Numbers display representative bands explained in the text.

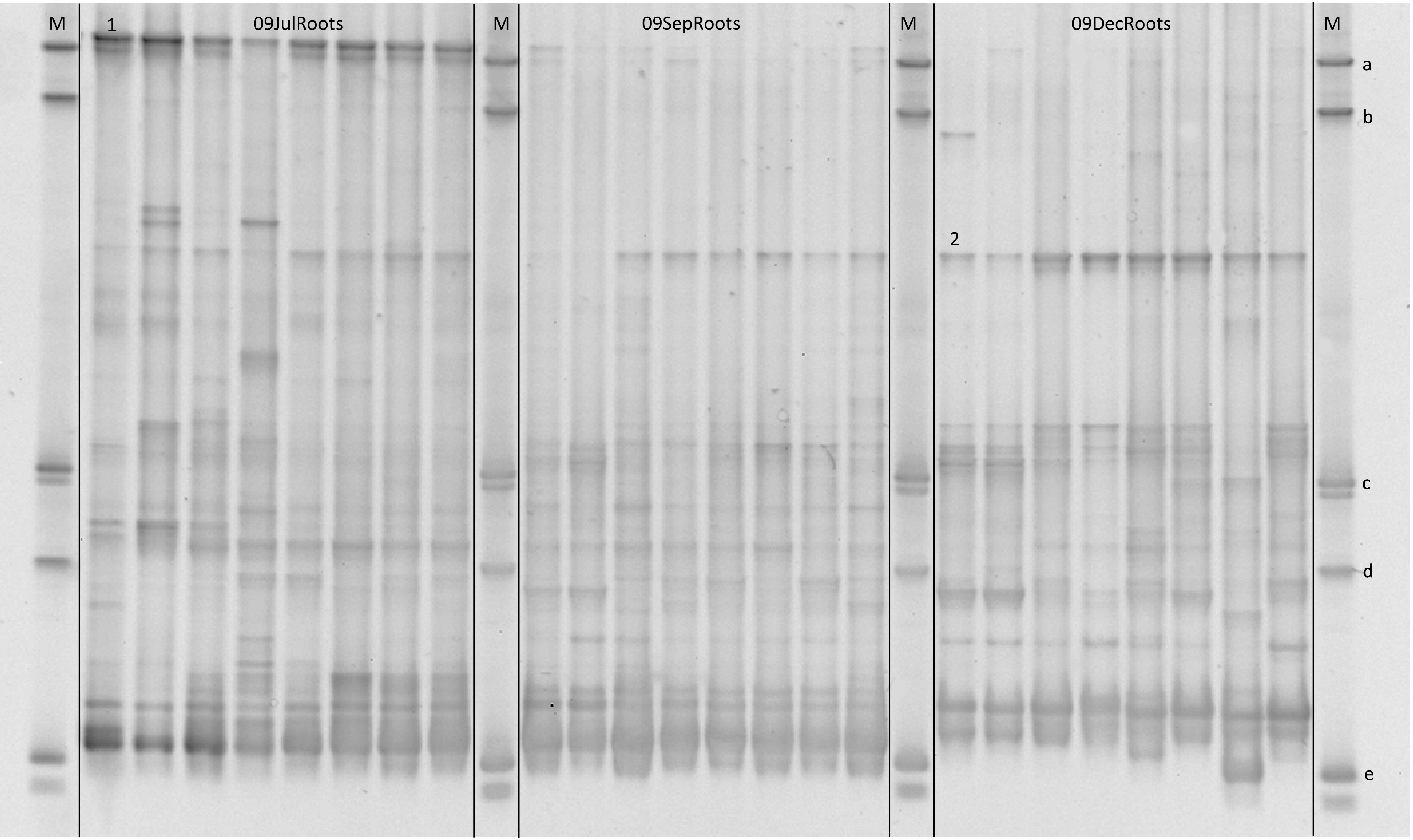

Fig B. DGGE results for fungal community structure of root compartment d for July, September and December 2009. Sample names contain the year (09 = 2009), abbreviated month (Jul = July, Sep = September, Dec = December). M = marker (a) *Mortierella cf. verticiellata, b) Mortierella cf. alpina, c) Rhodotorula sp., d) Exophiala sp, e) Verticillium cf. nigrescens*). Numbers display representative bands explained in the text.

Cocolin, L., L. Bisson and D. Mills (2000). "Direct profiling of the yeast dynamics in wine fermentations." FEMS Microbiology Letters **189**(1): 81-87.

Moll, J., K. Goldmann, S. Kramer, S. Hempel, E. Kandeler, S. Marhan, L. Ruess, D. Krüger and F. Buscot (2015). "Resource Type and Availability Regulate Fungal Communities Along Arable Soil Profiles." Microb Ecol: 1-10.
