# Supplementary material for: Spatial Distribution of Fungal Communities in an Arable Soil
Source: PLoS One. 2016 Feb 3;11(2):e0148130. doi: 10.1371/journal.pone.0148130 (PMC4740416; doi:10.1371/journal.pone.0148130)
Supplement: S1 Table — (PDF) [file pone.0148130.s006.pdf]

S1 Table. Soil properties of the field site from (Moll et al. 2015)

Horizon #	Depth [cm]	Bd [g cm^{-3}]	clay/silt/sand % (w/w)	pH ($CaCl_2$)	C_{org} [g kg^{-1}]	Total N [g kg^{-1}]	C/N	EOC * [µg g^{-1}]
Ap1	0-25	1.4±0.0	7.0/87.2/5.8	6.0±0.1	12.4±0.4	1.3±0.0	9.8	23.2±0.9
Ap2	25-37	1.6±0.0	7.1/87.8/5.0	6.2±0.1	6.9±1.2	0.8±0.1	9.2	17.2±0.7
Btw1	37-65	1.7±0.0	7.1/87.7/5.1	6.6±0.1	3.3±0.5	0.4±0.0	8.9	
Btw2	> 65	1.6±0.0	6.8/88.4/4.8	7.0±0.1	1.8±0.4	0.3±0.0	6.9	7.8±0.8

bd: bulk density; EOC: extractable organic carbon; # Classification according to KA5 (Ad-hoc-Arbeitsgruppe-Boden 2005); * values measured in 0-10 cm, 30-40 cm and 60-70 cm soil depth.

References

Ad-hoc-Arbeitsgruppe-Boden (2005). KA 5 (Bodenkundliche Kartieranleitung). Stuttgart, Germany, E. Schweizerbart'sche Verlagsbuchhandlung.

Moll, J, Goldmann, K, Kramer, S, Hempel, S, Kandeler, E, Marhan, S, Ruess, L, Krüger, D, Buscot, F (2015). "Resource type and availability regulate fungal communities along arable soil profiles." Microb Ecol: 1-10.
