# Supplementary material for: Spatial Distribution of Fungal Communities in an Arable Soil
Source: PLoS One. 2016 Feb 3;11(2):e0148130. doi: 10.1371/journal.pone.0148130 (PMC4740416; doi:10.1371/journal.pone.0148130)
Supplement: S2 Table — (PDF) [file pone.0148130.s007.pdf]

S2 Table. PerMANOVA results based on Bray-Curtis dissimilarities using abundance data for fungal community structure in relation to compartment for a) ITS data set, b) yeasts/dimorphics and c) AMF data set.

Environmental factors	Df	Sum Sq	Pseudo-F	R^2	P
a) ITS					
compartment	3	2.0331	2.7923	0.29521	**0.001**
residuals	20	4.8541		0.70479	
total	23	6.8872		1.00000	
b) Yeasts					
compartment	3	2.2023	3.4021	0.33789	**0.001**
residuals	20	4.3155		0.66211	
total	23	6.5178		1.00000	
c) AMF					
compartment	3	2.6488	4.1915	0.39825	**0.001**
residuals	19	4.0023		0.60175	
total	22	6.6511		1.00000	

Df - degrees of freedom; Sum Sq - sum of squares; Pseudo -F - F value by permutation, boldface indicates statistical significance with $P<0.05$, P-values based on 999 permutations (lowest P-value possible 0.001)
