# Supplementary material for: Correction: Spatial Distribution of Fungal Communities in an Arable Soil
Source: PLoS One. 2016 Apr 21;11(4):e0154290. doi: 10.1371/journal.pone.0154290 (PMC4839759; doi:10.1371/journal.pone.0154290)
Supplement: S1 File — (PDF) [file pone.0154290.s001.PDF]

PLOS ONE

RESEARCH ARTICLE

Spatial Distribution of Fungal Communities in an Arable Soil

Julia Moll[1,2]*, **Björn Hoppe**[1], **Stephan König**[3], **Tesfaye Wubet**[1,4], **François Buscot**[1,2,4], **Dirk Krüger**[1]

1 UFZ – Helmholtz Centre for Environmental Research, Department of Soil Ecology, Theodor-Lieser-Str. 4, 06120 Halle (Saale), Germany, **2** Institute of Biology, University of Leipzig, Johannisallee 21-23, 04103 Leipzig, Germany, **3** Julius Kühn-Institute - Federal Research Centre for Cultivated Plants (JKI) Institute for National and International Plant Health, Messeweg 11/12, 38104 Braunschweig, Germany, **4** German Centre for Integrative Biodiversity Research (iDiv) Halle – Jena - Leipzig, Deutscher Platz 5e, 04103 Leipzig, Germany

* julia.moll@ufz.de

CrossMark
click for updates

Abstract

Fungi are prominent drivers of ecological processes in soils, so that fungal communities across different soil ecosystems have been well investigated. However, for arable soils taxonomically resolved fine-scale studies including vertical itemization of fungal communities are still missing. Here, we combined a cloning/Sanger sequencing approach of the ITS/LSU region as marker for general fungi and of the partial SSU region for arbuscular mycorrhizal fungi (AMF) to characterize the microbiome in different maize soil habitats. Four compartments were analyzed over two annual cycles 2009 and 2010: a) ploughed soil in 0–10 cm, b) rooted soil in 40–50 cm, c) root-free soil in 60–70 cm soil depth and d) maize roots. Ascomycota was the most dominant phylum across all compartments. Fungal communities including yeasts and AMF differed strongly between compartments. Inter alia, *Tetracladium*, the overall largest MOTU (molecular operational taxonomic unit), occurred in all compartments, whereas *Trichosporon* dominated all soil compartments. Sequences belonging to unclassified Helotiales were forming the most abundant MOTUs exclusively present in roots. This study gives new insights on spatial distribution of fungi and helps to link fungal communities to specific ecological properties such as varying resources, which characterize particular niches of the heterogeneous soil environment.

OPEN ACCESS

Citation: Moll J, Hoppe B, König S, Wubet T, Buscot F, Krüger D (2016) Spatial Distribution of Fungal Communities in an Arable Soil. PLoS ONE 11(2): e0148130. doi:10.1371/journal.pone.0148130

Editor: Minna-Maarit Kytöviita, Jyväskylä University, FINLAND

Received: October 28, 2015

Accepted: January 13, 2016

Published: February 3, 2016

Copyright: © 2016 Moll et al. This is an open access article distributed under the terms of the Creative Commons Attribution License, which permits unrestricted use, distribution, and reproduction in any medium, provided the original author and source are credited.

Data Availability Statement: MOTU reference sequences are available from the International Nucleotide Sequence Database Collaboration portals under accessions HG935112-HG937193 and HG937194-HG937515, respectively.

Funding: The study was supported by the German Research Foundation (DFG) (http://www.dfg.de/) Research Unit FOR 918. The funders had no role in study design, data collection and analysis, decision to publish, or preparation of the manuscript.

Competing Interests: The authors have declared that no competing interests exist.

Introduction

Maize (*Zea mays* L.) is one of the most cultivated crops worldwide (http://www.fao.org). Although it strictly originated from Central America, this crop is actually cultivated in temperate Europe including Germany. In such crop monocultures fungi play a central role in plant diseases, but also for mobilization of nutrients from soil, primary production and decomposition processes [1–3]. Resources originating from plants with varying physical properties and chemical composition are heterogeneously distributed in soils. Slow decomposable plant material mainly exists in the topsoil, while rhizodeposits as readily available resources occur close to

the roots [4]. These two spheres represent distinct hot spot niches for soil fungi, and the distance to them structures fungal communities [4,5].

Apart these different intrinsic soil niches, the parts of plants growing in soils also represent habitats for fungi. Endophytic and symbiotic mycorrhizal fungi directly interact with their living host plants [6–9]. Maize is typically associated with arbuscular mycorrhizal fungi (AMF) to which they deliver carbon in exchange to mineral nutrients, mainly phosphorus [10]. The occurrence of this obligatory symbiotic fungal group is mainly expected in roots and in the rooted soil. However, they also explore the bulk soil and even litter layers where their diversity was underestimated for a long time [11]. In contrast, saprobic fungi are key biota in the decomposition of dead plant material. Members of the Dikarya (Asco- and Basidiomycota) are able to degrade recalcitrant substrates such as litter or plant leaves including lignocellulose compounds by secreting extracellular enzymes with strong oxidation potential [2,12]. Others, e.g. some zygomycetes rather live from more readily accessible substrates, such as root exudates [1]. Therefore, a resource depending distribution of saprobic fungi can be anticipated. Another ubiquitous existing fungal group in soils are the unicellular ascomycetous and basidiomycetous yeasts [13]. Despite their occurrence in diverse soil habitats yeasts are not known as primary degraders of complex polymers [14]. They are often designated as utilizers of low molecular weight compounds and thus are often present on roots and in the rhizosphere [14].

For arable soils, only a few studies have described that roots, rhizosphere and the bulk soil contain distinct fungal communities (e.g. [15,16]). At least, the spatial distribution of AMF is better investigated due to their importance as plant symbionts [17]. Moreover, although vertical distribution of microbial communities has received increasing attention [18–21] it is noteworthy that, to our knowledge, for total fungi in arable soils this assessment has solely been verified by Moll et al. [22]. Hence, an investigation of fungal taxa in different soil compartments including different soil depths and roots with taxonomic resolution of particular fungal groups is still missing and would improve our knowledge on fungal diversity related to particular soil niches.

The present study is a part of a broad arable field experiment installed to trace carbon fluxes originating from maize plants across trophic levels and link them to cross kingdom soil biodiversity [3]. We have recently shown by DNA fingerprints that different soil horizons contain specific fungal communities at the experimental field site [22]. The aim of the present study, which ran over two annual cycles in summer, autumn and winter 2009 and 2010/11, was to show which fungal taxa are characteristic of the following four compartments: a) the topsoil (0–10 cm) that is mostly influenced by recalcitrant plant material, b) the undisturbed rooted zone below the plough horizon (40–50 cm), c) the root-free soil (60–70 cm) that comprises the poorest C resources and d) the maize roots. We used a clone library/ Sanger sequencing approach on the ITS/LSU region within the Dikarya including their yeast forms, and on a greatly established part of the SSU region for targeting the AMF. Although sequencing depth in the present study does by far not reach those of next-generation sequencing investigations, the use of the complete highly variable ITS region in combination with the more conserved LSU region enable great MOTU assignment to taxa and longer reads provide important sequence information on fungal communities in arable soils.

The primary objective of this study was to assess the general hypothesis 1 that the community structure of general fungi, yeasts and AMF vary across the four compartments. Second we address the specific hypothesis 2 that especially yeasts and AMF are spatially distributed across the compartments according to their ecological roles.

Materials and Methods

Experimental design and sampling campaign

The experimental site was established in an arable field located at Göttingen-Holtensen (51°33´N, 9°53´E; 158 m NN, Lower Saxony, Germany). Field site and permission for performing the experiment were provided by the University of Göttingen. The dominant soil type is Luvisol and partly stagnic Luvisol [23]. Because of long-term agricultural use, two plough layers at 20 and 30 cm depth and a strong soil compaction below the second layer were discovered at the field site. Since April 2009 the investigated experimental plots have been cropped with maize (*Zea mays* L.). After harvest in autumn 2009 and 2010, aboveground maize material without cops was hackled and remained on plots (24 x 24 m). Basic soil properties are given in S1 Table and details about agricultural management (tillage and fertilizer practices) are described in Kramer et al. [3], Pausch and Kuzyakov [24] and Scharroba et al. [25]. Fungal communities were investigated over two annual cycles in 2009 and 2010. Summer sampling took place in July during vegetation growing season, autumn sampling in September shortly before harvest and winter sampling in December 2010 and January 2011 under fallow. Four replicated plots were considered for this study. Within one plot four compartments were sampled: a) soil from 0–10 cm for the investigation of the topsoil within the plough horizon, which is regularly enriched in litter, b) soil from 40–50 cm for rooted zone below plough horizon, c) soil from 60–70 cm for root-free soil in deeper horizons and d) maize roots (S1 Fig). For compartments a, b and c ten soil cores reaching 70 cm depth were randomly taken per plot. These soil profile samples were separated into 10 cm segments and soil from the same depth was thoroughly homogenized by hand. This resulted in one composite sample per plot and soil compartment, so that finally four samples originating from four plots represented each soil compartment. For root compartment d the root system from two plants per plot of the upper 20 cm soil were grubbed and approximately 20 fine roots of 2–3 cm length of each plant were randomly taken. All samples were immediately frozen on dried ice and stored at -80°C for later analyses.

A preliminary, but detailed DGGE (denaturing gradient gel electrophoresis) fingerprint analysis was performed to prove the heterogeneity of fungal communities between individual maize plots. It revealed homogenous distribution of fungal communities between these biological replicates. Procedure and exemplary results are shown in supplemental information (S1 File). Additionally, our recently published comprehensive study demonstrated, by analyzing F-ARISA fingerprints, that fungal community differences between vertical soil horizons were higher than within-group differences of biological replicates [22]. Based on these preliminary results we followed a procedure previously published by Renker et al. [26] to tackle variations in richness of fungal key species in studies geared towards high sample number. According to this procedure, DNA extractions and subsequent PCRs were done separately for each biological replicate. Afterwards, purified amplicons of the biological replicates were pooled for one cloning reaction per compartment and sampling date. Using this approach, Renker et al. [26] observed almost the same fungal diversity in comparison to the standard procedure of separate PCR and cloning for each replicate. As the four soil compartments were sampled six times over two annual cycles and Dikarya (ITS/LSU region) and AMF (SSU region) were assessed separately, 48 cloning reactions of pooled amplicons from biological replicates were conducted. A scheme of the experimental design is shown in S1 Fig.

DNA extraction

Total DNA was extracted from frozen soil samples separately for each replicated plot and soil compartment (4 plots x 3 soil samples x 6 sampling dates) with the PowerSoil DNA Isolation

Kit (MoBio Laboratories, Carlsbad, USA) as detailed in Scharroba et al. [25]. Roots were rinsed under running tap water and powdered by crushing under liquid nitrogen using pestle and mortar. The DNA of 120 mg powder of each root sample (2 plants for each replicated plot x 4 plots x 6 sampling dates) was then extracted with the DNeasy Plant Mini kit (Qiagen GmbH, Hilden, Germany) according to manufacturer's instructions.

PCR, cloning and sequencing

The primer pair ITS1F and LS2r was used to amplify the ITS and adjacent LSU (D1) region (together called ITS data hereafter) of the fungal rDNA [27,28]. The PCR on each DNA extract was performed in separate 20 μl reaction mixtures containing 4 μl FIREPol 5x Master Mix (Solis BioDyne, Tartu, Estonia), 10 μM of each primer and approximately 20 ng template DNA. Cycling conditions included an initial denaturation step at 95°C for 5 min followed by 35 cycles at 95°C for 40 s, 54°C for 30 s and 72°C for 2 min. Elongation was completed with a final step of 72°C for 10 min. Amplicons were purified using the peqGOLD Cycle-Pure Kit (PeqLab GmbH, Erlangen, Germany). Afterwards, replicated PCR products for each soil compartment were pooled before ligation as explained above. Cloning was done with pGEM-T Vector System (Promega GmbH, Mannheim, Germany) and *Escherichia coli* JM109 according to the manufacturer's instructions. Approximately 90 clones per clone library were PCR screened for the insert by re-amplification of the insert with primers M13F and M13R and the following PCR-conditions: 95°C for 5 min, 32 cycles of 95°C for 40 s, 54°C for 30 s and 72°C for 60 s and a final elongation step of 72°C for 10 min. Amplicons were checked on 1% agarose gels under UV light. Clones with insert were purified with ExoSAP-IT (USB Corporation, Cleveland, Ohio, USA) and then used in cycle sequencing in both directions using M13 PCR primers and the Big Dye Terminator Cycle Sequencing Reaction Kit v.3.1 (Applied Biosystems, Life Technologies, Darmstadt, Germany). After an ethanol precipitation sequencing was completed on an ABI 3730xl DNA Analyzer (Applied Biosystems).

A nested and touchdown PCR strategy was used to amplify the NS31-AM1 fragment of the SSU rDNA for AMF. The first PCR was performed with the primer pair GlomerWT0/ Glomer1536 [29] and an initial denaturation for 30s at 98°C followed by: (1) 5 cycles of 94°C for 30 s, 60–55°C for 30 s (−1°C per cycle) and 72°C for 1 min; and (2) 25 cycles of 94°C for 30 s, 55°C for 30 s and 72°C for 1 min with a final extension step of 5 min. The second PCR made use of primer NS31 [30] and reverse primers AM1a and AM1b [31] to obtain all groups of Glomeromycota. Products of the first PCR were diluted 1:10 and then used for the second PCR, which was performed with the following conditions: 30s at 98°C, 30 cycles of 94°C for 30 s, 55°C for 30 s and 72°C for 1 min and a final extension of 5 min at 72°C.

Cloning and sequencing of AMF amplicons was conducted using the Perfect PCR Cloning Kit (5 Prime, Hamburg, Germany) and *Escherichia coli* TOP10 cells (Invitrogen, Life Technologies, Darmstadt, Germany) otherwise as described above. Because of the expected lower diversity of AMF, only 32 clones per clone library were sequenced.

Sequence analyses

Forward and reverse strands were assembled using Sequencher 4.10 (Genecodes, Ann Arbor, MI, USA), contigs were manually edited and consensus sequences of these contigs (after removal of vector and primer remnants) were maintained in BioEdit (Ibis Biosciences, Carlsbad, CA, USA) as database. Building of MOTUs (molecular operational taxonomic units) and de-replication were performed using the BLASTclust module of the NCBI-BLAST package ported at http://toolkit.tuebingen.mpg.de/blastclust specifying as coverage 80% and as identity threshold 97% for general fungal ITS and 98% for AMF SSU rDNA. The identification of the

MOTU reference sequence was done by BLASTn query against the DDBJ nucleotide sequence database using standard settings but excluding environmental sequences and targeting the plant division that contains all fungi in that database. The ten best hits were recorded. The MOTUs were immediately given the exact name of the best hit without any conflict between the top hits if the 'Expect value' was 0, and the score approximately two times the length of the query sequence. In all other cases, BLASTn was repeated against the complete NCBI GenBank version of the international nucleotide database due to its visual display of sequence coverage and to exclude non-Fungi, and queries that hit database sequences from different orders or higher in different parts across their length were excluded from further analyses for being potential chimeras. Higher taxonomic groups followed NCBI taxonomy and Index Fungorum (www.indexfungorum.org). For SSU AMF data set all non-AMF sequences were excluded from further sequence analyses. To test whether the number of sequenced clones adequately represented fungal populations, rarefaction curves were calculated for each compartment using the software PAST [32].

Fungal community sizes separated by compartment were displayed using a 4-way Venn diagram (modified after creation in Venny at http://bioinfogp.cnb.csic.es/tools/venny/index.html, Oliveros 2007). Subsequently, a heatmap for the sum abundance of fungal sequences with color ranges defined in MS Excel was overlaid onto the Venn sectors. Piecharts with ecological information based on current taxonomic literature were done in OpenOffice Calc 3.3.0. IBM ManyEyes (word cloud function) was used to show the largest MOTUs per compartment in font sizes reflecting relative MOTU sizes. Subsequent combination of these graphical elements, color definition and editing of colors between different graphics was done using Visual Color Picker v. 2.6 (NOVOSIB Software Co.), MS Paint and the Corel Draw Graphics Suite X3 (Corel Corporation, Ottawa, Canada).

Statistical analyses

Statistical analyses were performed with R version 3.0.1. [33]. All multivariate analyses on fungal community structure were assessed using permutational multivariate analysis of variance (perMANOVA) based on Bray-Curtis distance using the function 'adonis' in the package 'vegan' [34,35]. An initial multivariate statistical analysis (perMANOVA) on fungal community structure related to compartments and sampling dates revealed highly significant differences between compartments ($F = 0.29521$, $p = 0.001$) and marginally significant differences between sampling dates ($F = 0.21081$, $p = 0.052$). Due to complex data set, the study mainly focuses on space specific results comparing fungal communities between the four different compartments. In this case, the six different sampling dates served as replicates. We analyzed space differentiation of (1) the overall fungal communities, (2) yeast and dimorphic fungi (with yeast stages) and (3) AMF, whereas the latter is a separate dataset independent of the general fungal data due to primer specificities.

Detailed perMANOVA results are given in S2 Table. PerMANOVA does not provide a graphical output, therefore multivariate results were visualized using Bray-Curtis distance based non-metric multidimensional scaling (NMDS) biplots. For better clarity p-value ($^{*} = P < 0.05$; $^{**} = P < 0.01$; $^{***} = P < 0.001$) and R^2 of each perMANOVA were directly included in the NMDS plots. R^2 ranges between 0 and 1. Hence, the relative proportion of variation of each factor is directly visible. Multivariate statistical results in this study are based on sequence counts. To ensure that results are consistent analyses were also done i) excluding singletons (SMOTUs), ii) relative abundance data and iii) presence/absence data. All analyses revealed comparable results, which enable us drawing reliable conclusions.

Results

Fungal community structure (ITS data set)

A total of 2,082 quality-controlled sequences were obtained from 24 ITS clone libraries in the experiment (INS accession numbers HG935112-HG937193). Rarefaction curves did not reach saturation, indicating that further sequencing would have revealed additional MOTUs (S2 Fig). Nevertheless, curves pass the point of linearity, indicating that most prevalent fungi were likely identified. 313 MOTUs were formed, 154 of these were singletons (S4 Fig). Ascomycota were the most dominant group with 68.3% of the sequences (68.4% of the MOTUs), followed by 2.4% (25.6% of the MOTUs) Basidiomycota, 8.5% (4.2% of the MOTUs) Mucoromycotina and less than 1% each Glomeromycota (1.6% of the MOTUs) or fungi of unknown taxonomic affiliation (0.3% of MOTUs) (S3A and S3B Fig).

As displayed in Fig 1 Pezizomycotina was the most abundant subphylum among the Ascomycota, where it accounted for 47–93% of the relative abundance in different compartments. Only in compartment c in September 2009 (09SepS70) the most dominant subphylum was represented by the basidiomycetous Pucciniomycotina (42%). Among Basidiomycota, members of the Agaricomycotina represented the highest dominance across samples. Within the ITS data set Glomeromycotina were only detected during vegetation growing season in compartments a, b, and d.

Multivariate statistical analysis (perMANOVA) over all sampling dates and its visualization by NMDS biplot showed that fungal community structure significantly differed between compartments ($p = 0.001$), mainly recognizable by a differentiation between the three soil compartments a, b, c and the roots d (Fig 2A).

Compartment differentiation and fungal key species. Most sequences found with ITS primers were identified as saprobic fungi and belonged to MOTUs that were shared between compartments. Approximately 50% of sequences were from MOTUs present in all four compartments (Fig 3, labeled as abcd). The overall largest MOTU (*Tetracladium*), *Exophiala* (MOTU 8) as well as *Chaetomium* (MOTU 5) were commonly found in all compartments (Fig 4). *Davidiella* (MOTU 2), teleomorphic to *Cladosporium*, were also present in all compartments but mainly in the deepest soil layer c, whereas *Verticillium* (MOTU 6) were mainly observed in the upper soil horizons a and b (Fig 4). Approximately 11% of sequences were shared between the three soil compartments (Fig 3, labeled as abc), from which more than two thirds were identified as saprobes. The largest MOTUs of this compartment overlap were affiliated to the basidiomycetous yeast *Trichosporon* (MOTU 3) dominating the soil compartments followed by *Cryptococcus* (MOTU 21).

Only a small fraction of MOTUs accounted exclusively for 3, 3, 6 and 6% of the relative abundances for the four compartments a, b, c and d, respectively. For instance, MOTU 16 belonging to *Microdochium*, MOTU 17 and 22 (uncultured Helotiales), MOTU 19 (*Mycochaetophora*) and MOTU 24 (*Periconia*) were typically detected from roots (Fig 4).

Arbuscular mycorrhizal fungi (AMF) were not only detected in roots (Fig 3, labeled as d), but also in the overlap between roots and the topsoil compartment (labeled as ad) and between roots and the rooted soil compartment (labeled as bd). As the AMF were generally rarely found with the ITS primers, this fungal group was additionally investigated using specific primers.

Yeasts. Sixty-five different yeast-like or dimorphic fungal MOTUs were detected from the ITS data set. Multivariate statistical analyses (perMANOVA) on this yeast community structure revealed significant differences in their distribution between soil compartments ($p = 0.001$). The NMDS shows that root compartment d significantly differed from the soil compartments a-c (Fig 2B). In soil compartments a, b and c 13–69% of ITS sequences were identified as yeast-like fungi, whereas in the root compartment d yeast proportions of 4-13%

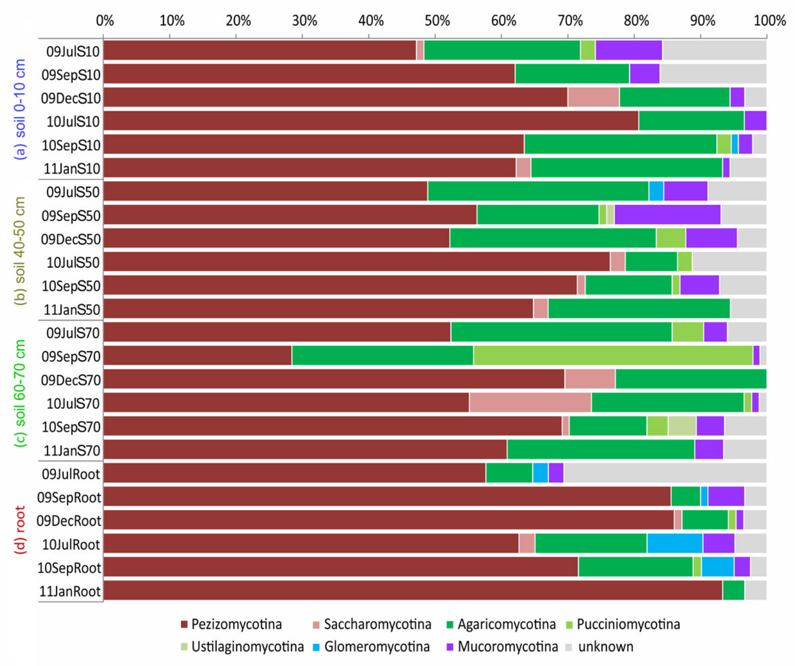

Fig 1. Fungal community structure of the ITS dataset by subphyla across the four compartments a-d for all sampling dates. Sample names contain the year (09 = 2009, 10 = 2010, 11 = 2011), abbreviated month (Jul = July, Sep = September, Dec = December, Jan = January) and depth (S10 = Soil 0–10 cm, S50 = Soil 40–50 cm, S70 = Soil 60–70 cm).

doi:10.1371/journal.pone.0148130.g001

were detected (Fig 5). We found prominent yeasts (e.g. *Trichosporon* MOTU 2, *Cryptococcus* MOTU 13) all over the year. In compartment c of September 2009 almost 70% of fungal sequences (50% of MOTUs) were identified as yeasts (Fig 5). Mainly *Rhodotorula* (MOTU 25) and *Cryptococcus* (MOTU 26) contributed to this high proportion (S4 Fig). This is consistent with our preliminary DGGE results for this compartment showing three very dominant phylotypes 5–7 including one dominant phylotype 5 on the same running position as the reference band c *Rhodotorula* sp. (S1 File).

Arbuscular mycorrhizal fungal communities (SSU data)

A total of 322 quality-controlled sequences (accession numbers HG937194-HG937515) belonging to Glomeromycota were obtained from AMF clone libraries. Using specific SSU primers AMF could be detected across all compartments, although compartment c of December 2009 comprised no AMF MOTU of 32 quality-checked sequences (sample 09DecS70 in Fig 6). 17 unique MOTUs were detected, 7 of these were singletons (SMOTU). MOTUs were identified as belonging to four orders Archaeosporales (8,4% of sequences), Glomerales (72,4% of sequences), Diversisporales (1,6% of sequences) and Paraglomales (17,7% of sequences). In comparison, the ITS sequence dataset contained only 5 MOTUs, belonging to Glomerales, Paraglomerales, Diversisporales. The occurrence of the MOTUs across the compartments a-d for all sampling dates is shown in Fig 6 and the order composition in S3C and S3D Fig

AMF community structure significantly differed between compartments (Fig 2C). The first NMDS axis mainly separated the AMF communities of compartments a and d from those of b and c. For instance, MOTU 5 (*Archaeospora* sp.) was only found in the topsoil a and in roots d. In contrast, MOTU 1 (*Glomus* sp.) was exclusively present in the two soil compartments b and

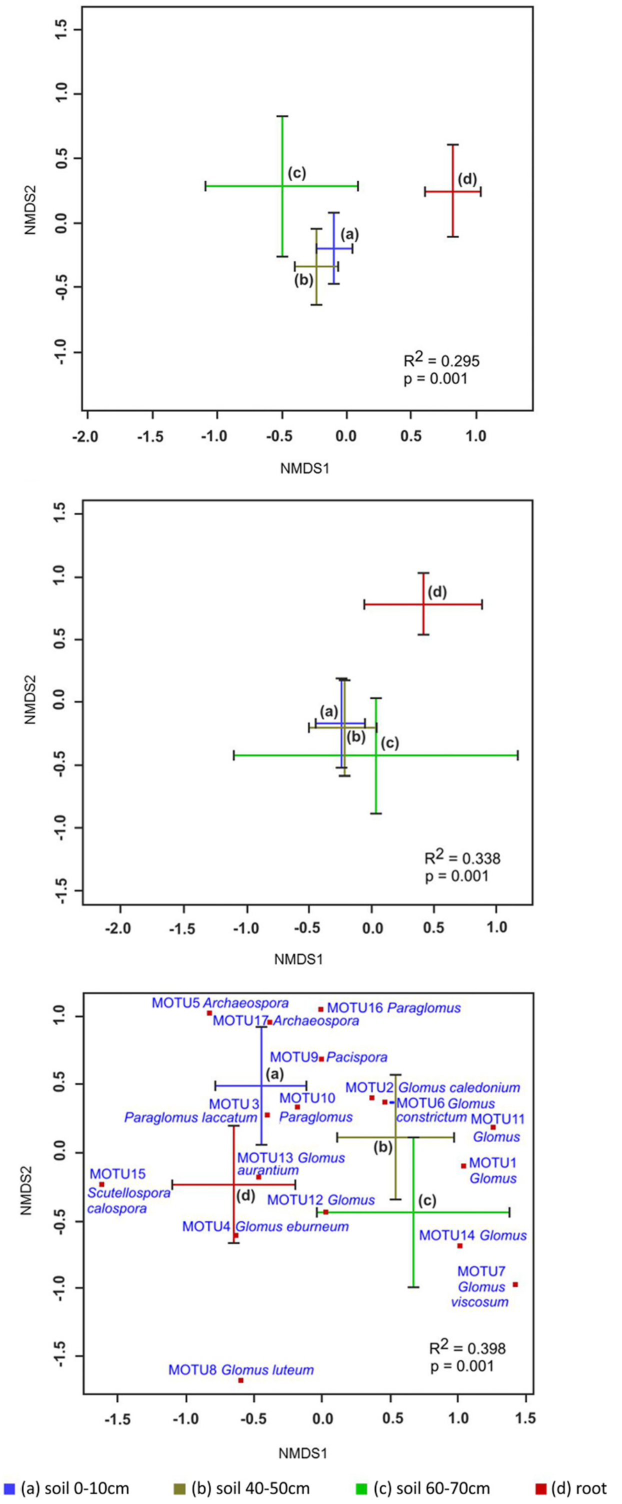

Fig 2. Two-dimensional non-metric multidimensional scaling (NMDS) biplots based on Bray-Curtis distances displaying differences of A) the total fungal community in the ITS dataset, B) yeasts and dimorphics in the ITS dataset and C) AMF from SSU rDNA dataset in relation to compartment. Bars represent one standard deviation along both NMDS axes. Only C is labeled with individual MOTUs for readability. Stress value was for A) 0.15, for B) 0.16 and for C) 0.12.

doi:10.1371/journal.pone.0148130.g002

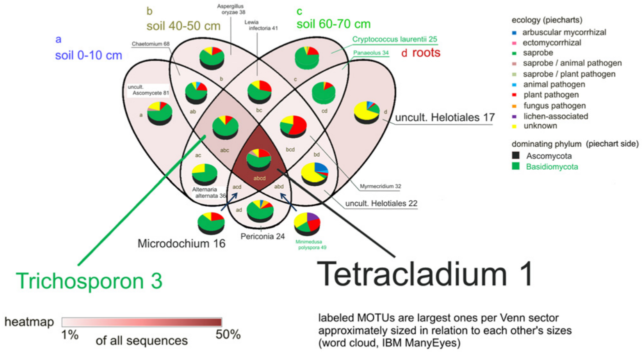

Fig 3. Fungal community. Venn diagram showing ratios of sequences occurring in (a) soil 0-10 cm, (b) soil 40–50 cm, (c) soil 60–70 cm and (d) roots and in their overlaps. Piecharts indicate the ecological composition of the compartmental communities. Relative contribution of compartments or their overlaps to the total number of sequences is also given by a heat map. Within each compartment/overlap, the largest MOTUs are shown. The size of MOTU labels within each compartment/overlap reflects relative sizes by sequence membership (from IBM ManyEyes), green MOTU labels = Basidiomycota, black = Ascomycota.

doi:10.1371/journal.pone.0148130.g003

c below the plough layer (Figs 2C and 6). *Paraglomus laccatum* (MOTU 3) was a dominant member of the AMF communities in compartments a, b and d, but was never detected in the root-free soil c. Only two non-specialized MOTUs belonging to the genera *Glomus* (MOTU 2 and 4) were observed across all compartments.

Discussion

Fungal community structure (ITS data)

Different soil ecosystems under forests, grasslands or arable sites exhibit specific fungal communities. For instance, in forest soils Basidiomycota often dominate the fungal community [36–38]. One explanation could be that the decomposition of more recalcitrant materials found in forest soils is mainly attributed to fungi from the Agaricomycotina (Basidiomycota). As woody debris are missing in ecosystems with an annual plant cropping, dominance of Ascomycota (mainly Pezizomycotina) observed in this study is not surprising. Therefore, our results are in line with previous studies on fungi in arable soils [39–42].

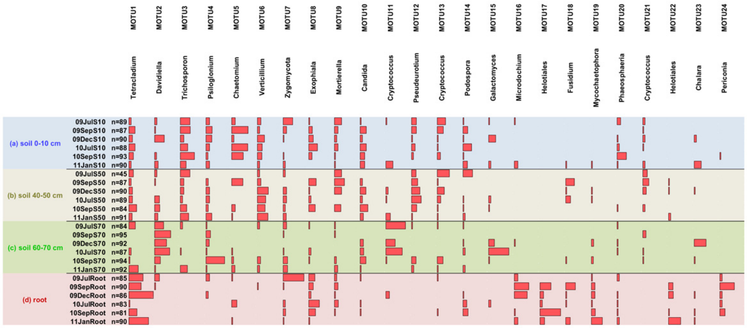

Fig 4. Excel in-cell chart of most abundant fungal MOTUs (≥ 25 sequences) and their distribution in (a) soil 0-10 cm, (b) soil 40–50 cm, (c) soil 60–70 cm and (d) roots. Red bars represent relative abundance of respective MOTU in the sample, smallest bars are 1 sequence. Sample names contain the year (09 = 2009, 10 = 2010, 11 = 2011), abbreviated month (Jul = July, Sep = September, Dec = December, Jan = January) and depth (S10 = Soil 0–10 cm, S50 = Soil 40–50 cm, S70 = Soil 60–70 cm). n = number of quality checked sequences.

doi:10.1371/journal.pone.0148130.g004

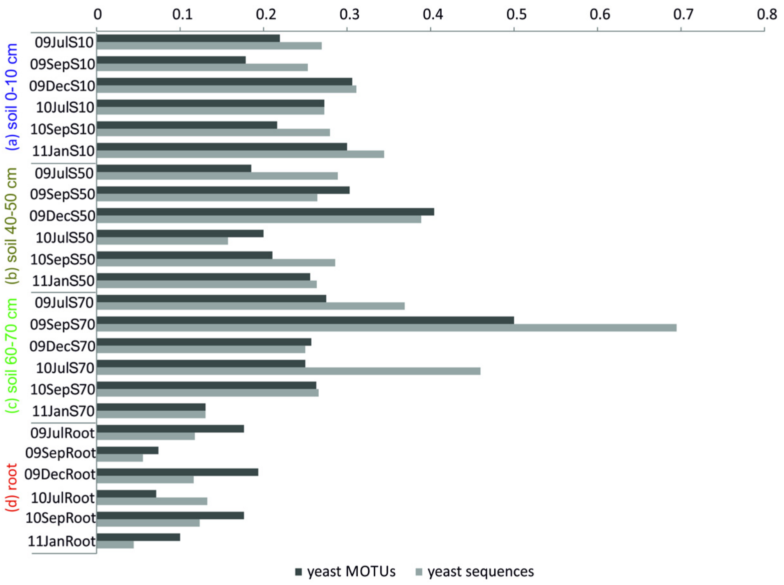

Fig 5. Ratio of yeast/dimorphic sequences and MOTUs to complete ITS dataset across the four compartments a-d for all sampling dates. Sample names contain the year (09 = 2009, 10 = 2010, 11 = 2011), abbreviated month (Jul = July, Sep = September, Dec = December, Jan = January) and depth (S10 = Soil 0–10 cm, S50 = Soil 40–50 cm, S70 = Soil 60–70 cm).

doi:10.1371/journal.pone.0148130.g005

Compartment differentiation and fungal key species. Fungi are known to be influenced by abiotic factors such as soil structure, pH or oxygen availability and biotic factors such as resource type and availability [22,43–46] and therefore these factors structure fungal niche space. In accordance with hypothesis 1 the present study shows a distinct differentiation of fungal communities across compartments. Fungal generalists that were detected across all compartments seem to be able to inhabit niches with different ecological conditions, e.g. varying resource availabilities or the existing microclimate. Exemplary generalists at the experimental field site were MOTU 7 (unclassified Zygomycota), MOTU 8 (*Exophiala*), and MOTU 10 (*Candida*). The overall most frequent MOTU (*Tetracladium*) also falls into this category. This

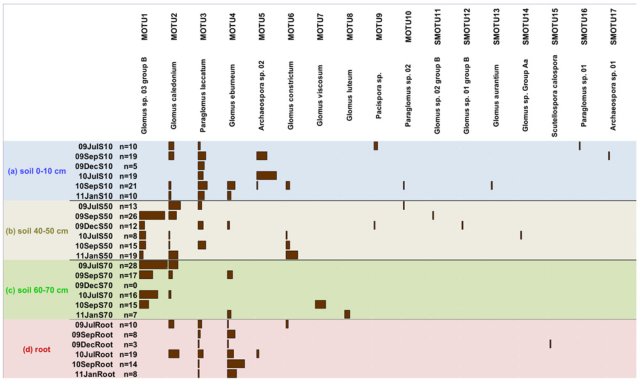

Fig 6. SSU rDNA AMF MOTUs and their distribution in (a) soil 0-10 cm, (b) soil 40–50 cm, (c) soil 60–70 cm and (d) roots. SMOTU = singleton MOTU. Sample names contain the year (09 = 2009, 10 = 2010, 11 = 2011), abbreviated month (Jul = July, Sep = September, Dec = December, Jan = January) and depth (S10 = Soil 0–10 cm, S50 = Soil 40–50 cm, S70 = Soil 60–70 cm). n = number of quality checked AMF sequences.

doi:10.1371/journal.pone.0148130.g006

genus is known as ubiquitously distributed in soils as well as for endophytic or aquatic lifestyle [16,39,47–50]. MOTU 9 (*Mortierella*) belongs to zygomycetes; members of this group are so called sugar fungi and are often associated with the utilization of easily accessible carbon such as Glucose [1,2]. Therefore, their presence would be expected mainly in the rhizosphere or rooted zone. Here, *Mortierella* did not show a clear compartmentalization, suggesting appropriate ecological conditions for this MOTU in the range of examined soil habitats. *Trichosporon* (MOTU 3) dominated the three soil compartments a, b and c. Although members of this genus have been reported for their capability to degrade cellulose and to enhance plant growth [14,51], in this study it was not detected in roots. Nevertheless, due to the ability to exploit all investigated soil compartments (a, b and c) and its broad distribution in agricultural, forest and grassland soils of different climatic zones, *Trichosporon* (MOTU 3) can also be defined as a generalist [52–56].

Despite observed generalists, multivariate analysis displayed main separation of fungal communities in the root compartment from those of the soil compartments. Hence, roots likely provide a distinct niche for specialized fungi. For instance, MOTU 17 and 19 (unclassified Helotiales) as well as MOTU 16 (*Microdochium*), 19 (*Mycochaetophor*a) and 24 (*Periconia*) were highly abundant in roots. Those fungal taxa likely depended on easily accessible substrates from the root, thrive on recalcitrant dead plant roots or colonize roots as they were almost not observed in the soil compartments.

Within the three soil compartments (a, b and c) distinct fungal communities could also be determined. Fungal communities of compartments a and b, which were associated with plant residues and root exudates, were mainly separated from those of compartment c, which were furthest away from plant resources (Fig 2A). These changes in fungal community structure correspond quite well to the changing amount of available resources with increasing depth, as a predictable reduction in total and extractable organic carbon and total nitrogen was observed at the experimental field site (S1 Table).

Interestingly, assessing the ITS region the same AMF MOTUs were detected in roots d and in the overlap between roots and the topsoil (ad) or roots and the rooted zone (bd). These results emphasize the importance of AMF in the exchange and link of carbon between plant and soil [10,57]. Furthermore, the fact, that with general fungal primers AMF sequences were not detected in any winter sample and in any 60–70 cm soil depth sample, suggests a clear temporal and spatial compartmentalization for this functional group (hypothesis 2).

Yeasts. Filamentous fungi have received interest due to their ability to bridge distances and to explore and exploit new soil habitats via hyphal growth [58,59]. Nevertheless, unicellular yeasts are common soil inhabiting fungi in arable and also maize planted soils [13,15,54]. In the present study yeasts were among the most dominant fungal generalists obviously able to inhabit different compartments (e.g. *Trichosporon*, *Candida*, *Exophiala*), although a main differentiation between root and the three soil compartments could be determined (hypothesis 1).

Especially the basidiomycetous *Cryptococcus* was strongly represented across all samples, which is in line with other studies assessing fungal diversity in arable soils [54,60]. Gomes et al. [15] reported such yeasts mainly from senescent *Zea mays* rhizosphere, while we observed prominent yeasts (e.g. *Trichosporon* MOTU 3, *Cryptococcus* MOTU 13) all over the year, representing different plant developmental stages. Indeed, yeasts have been described to thrive on various substrates including recalcitrant and aromatic compounds [14]. Nevertheless, they are often observed near to plant roots presumably utilizing cellulosic and labile compounds percolating into the soil or even to inhabit roots themselves [14,61–63]. Concerning our hypothesis 2, we would have expected to detect yeasts especially in the root compartment. In contrast, yeast ratio was lowest in/on roots compared to the three soil compartments. Expanding the present investigation by additionally analyzing rhizosphere soil may help to explain these

outcomes. Likewise, Xu et al. [16] reported that yeasts such as *Trichosporon* and *Cryptococcus* were almost exclusively present in the rhizosphere and bulk soil but not in roots.

Arbuscular mycorrhizal fungal communities (SSU data)

The diversity of AM fungal communities is known to be influenced by abiotic and biotic factors such as soil properties, management practices, numbers of potential host plant species and host development stages [7,64–66]. Spatial- and temporal-based differences in mycorrhizal fungal communities have been recently reviewed by Bahram et al. [17], who greatly demonstrated that vertical variation exceed horizontal and temporal aspects. For AMF in arable soils temporal and vertical variations have been mainly investigated by assessing spores [67–69]. For instance, Tian et al. [70] observed a stronger effect of soil depth than maize management practice on AMF communities. However, to our knowledge only two studies explored vertical AMF diversity using molecular tools [71,72]. Results are in line with our AMF data set showing distinct communities across depth. Moreover, according to hypothesis 1, we detected differentiation across compartments, including only two general MOTUs inhabiting all compartments. These two generalists (MOTU 2 and 4) belonged to the *Glomeraceae*. Members of this family inhabit a wide range of terrestrial ecosystems and also different niches even on a fine-scale level in arable soils [9,73]. The adaptability of several taxa could be attributed to their fast growth rate, to efficient hyphal fusion mechanisms and to a large abundance of intra-radical hyphal biomass, factors that likely favor their distribution in disturbed arable soils [74].

We observed strong spatial variations with topsoil and root (compartment a and d) communities mainly separated from those of the two deeper soil layers (compartment b and c). As root samples were taken from upper soil in 0–20 cm and as about 50% of the root biomass were distributed in the upper 10 cm of the Ap horizon [75], it is obvious that topsoil and roots exhibit similar communities of this symbiotically living group. Hence, these results support hypothesis 2, that AMF are distributed according to their ecological role. Complementary to that, MOTU 3 (*Paraglomus* sp.) were detected in all compartments associated with the host (rooted soil of compartment a and b and roots) but not in the root-free soil, suggesting that this species was an active key symbiont able to link different ecological niches at the investigated field site. In contrast, MOTU 1 (*Glomus* sp.) was absent within the topsoil and roots (compartment a and d). One reason could be that the topsoil is not only the location with the highest resource input from aboveground, but is also directly affected by agricultural practices such as fertilization and tilling. Such disturbances intensively affect AMF diversity and biomass [9,76,77]. The fact that MOTU 1 was absent in the potential host plant (root compartment d) could illustrate the consideration that some AMF appearing in the soil await their recruitment by the host plant [11], which in turn likely explains potential high AMF diversity in the bulk soil.

Here, conclusions on the habitat distribution of fungal communities were drawn mainly without respect to temporal variations. We are aware that especially for AMF, further investigations including considerations of separate sampling dates are needed. This becomes obvious at winter sampling when no living host plant was available and is supported by the result that compartment c of December 2009 comprised no AMF MOTU of 32 quality-checked sequences (sample 09DecS70).

In conclusion, the present study provides a description of the most dominant fungal species in an arable soil including spatial fine-scale investigations. Some general conclusions on fungal key species can be drawn: (1) major fungal MOTUs occurred over all seasons; (2) all investigated fungal groups (overall fungi, yeasts and AMF) showed strong differentiation across

compartments; (3) considering the ITS data, communities mainly varied between the soil and root; whereas (4) considering the SSU data, AMF communities of roots and topsoil were mainly separated from communities of the two deeper soil layers.

Our results indicate the presence of distinct niches for microbial life in arable soils and show the importance of considering different soil habitats for fungal diversity studies, which will allow linking diversity to specific ecological processes. However, this study gives only an overview on prominent fungi due to limited number of Sanger sequences, although those long ITS/LSU reads provide important fungal sequence information. A prospective is the use of an approach allowing handling with more samples and deeper sequencing e.g. using next generation sequencing platforms.

Supporting Information

S1 Fig. Experimental design displayed for one sampling date. Sampling took place in summer, autumn and winter over two annual cycles (2009 and 2010/2011). Four replicated plots were sampled. Within one plot four compartments a) 0–10 cm, b) 40–50 cm and c) 60–70 cm soil depth and d) maize roots were analyzed. Within one plot each of three soil compartments a-c consisted of soil from ten soil cores of respective depth, so that finally four replicated samples from four plots represented each soil compartment. For sampling the root compartment d, two maize plants per plot were grubbed and approximately 20 fine roots were randomly taken. DNA extraction of all sampled compartments was performed separately for each plot. Genomic DNA was used 1) for the amplification of the ITS/LSU region and 2) for the amplification of the SSU region. Purified amplicons of the biological replicates were pooled and used for one cloning reaction for each compartment. Finally, 96 clones for the ITS/LSU region and 32 clones for the SSU region were screened by Sanger sequencing.
(TIF)

S2 Fig. Rarefaction curves of each compartment for A) ITS and B) AMF data set.
(TIF)

S3 Fig. Piecharts of overall fungal community composition. A) Distribution among MOTUs for ITS dataset by phylum, B) distribution among sequences for ITS dataset by phylum, C) distribution among MOTUs for AMF dataset by order and D) distribution among sequences for AMF dataset by order.
(TIF)

S4 Fig. Overview on complete ITS fungal MOTUs across the four compartments a-d without singletons. Red bars represent relative abundance of respective MOTU in the sample, smallest bars are 1 sequence. The sample names to the left contain the year (09 = 2009, 10 = 2010, 11 = 2011), abbreviated month and depth, number of sequences. Sample names contain the year (09 = 2009, 10 = 2010, 11 = 2011), abbreviated month (Jul = July, Sep = September, Dec = December, Jan = January) and depth (S10 = Soil 0–10 cm, S50 = Soil 40–50 cm, S70 = Soil 60–70 cm), n = number of quality checked sequences.
(PDF)

S1 File. Supporting information on fungal community structure analysis by denaturing gradient gel electrophoresis (DGGE) fingerprints.
(PDF)

S1 Table. Soil properties of the field site.
(PDF)

S2 Table. PerMANOVA results on fungal community structure in relation to compartment for a) ITS data set, b) yeasts/dimorphics and c) AMF data set.
(PDF)

Acknowledgments

We thank Olaf Butenschoen for management at the field site and the many field work helpers. Kezia Goldmann, Melanie Günther and Renate Rudloff are gratefully acknowledged for their technical assistance during laboratory work. Andrey Yurkov is thanked for identifying the yeast taxa in our data and Stefan Hempel for visualization of NMDS plots.

Author Contributions

Conceived and designed the experiments: FB DK JM. Performed the experiments: JM. Analyzed the data: JM SK DK. Contributed reagents/materials/analysis tools: TW FB DK. Wrote the paper: JM DK. Contributed to design and formatting of figures: BH DK JM. Edited the manuscript: JM BH SK TW DK FB.

References

1. de Boer W, Folman LB, Summerbell RC, Boddy L. Living in a fungal world: impact of fungi on soil bacterial niche development. FEMS Microbiol Rev. 2005; 29: 795–811. PMID: 16102603
2. van der Wal A, Geydan TD, Kuyper TW, de Boer W. A thready affair: linking fungal diversity and community dynamics to terrestrial decomposition processes. FEMS Microbiol Rev. 2013; 37: 477–494. doi: 10.1111/1574-6976.12001 PMID: 22978352
3. Kramer S, Marhan S, Ruess L, Armbruster W, Butenschoen O, Haslwimmer H, et al. Carbon flow into microbial and fungal biomass as a basis for the belowground food web of agroecosystems. Pedobiologia. 2012; 55: 111–119.
4. Kuzyakov Y, Blagodatskaya E. Microbial hotspots and hot moments in soil: Concept & review. Soil Biol Biochem. 2015; 83: 184–199.
5. Berg G, Smalla K. Plant species and soil type cooperatively shape the structure and function of microbial communities in the rhizosphere. FEMS Microbiol Ecol. 2009; 68: 1–13. doi: 10.1111/j.1574-6941.2009.00654.x PMID: 19243436
6. Pan JJ, Baumgarten AM, May G. Effects of host plant environment and *Ustilago maydis* infection on the fungal endophyte community of maize (*Zea mays*). New Phytol. 2008; 178: 147–156. doi: 10.1111/j.1469-8137.2007.02350.x PMID: 18194146
7. Verbruggen E, Van Der Heijden MG, Weedon JT, Kowalchuk GA, Roling WF. Community assembly, species richness and nestedness of arbuscular mycorrhizal fungi in agricultural soils. Mol Ecol. 2012; 21: 2341–2353. doi: 10.1111/j.1365-294X.2012.05534.x PMID: 22439851
8. Brito I, Goss MJ, de Carvalho M, Chatagnier O, van Tuinen D. Impact of tillage system on arbuscular mycorrhiza fungal communities in the soil under Mediterranean conditions. Soil Till Res. 2012; 121: 63–67.
9. Borriello R, Lumini E, Girlanda M, Bonfante P, Bianciotto V. Effects of different management practices on arbuscular mycorrhizal fungal diversity in maize fields by a molecular approach. Biol Fertil Soils. 2012; 48: 911–922.
10. Smith SE, Read DJ. Mycorrhizal symbiosis. 3 ed. London, UK: Academic Press; 2008.
11. Buscot F. Implication of evolution and diversity in arbuscular and ectomycorrhizal symbioses. J Plant Physiol. 2015; 172: 55–61. doi: 10.1016/j.jplph.2014.08.013 PMID: 25239593
12. Baldrian P, Voriskova J, Dobiasova P, Merhautova V, Lisa L, Valaskova V. Production of extracellular enzymes and degradation of biopolymers by saprotrophic microfungi from the upper layers of forest soil. Plant Soil. 2011; 338: 111–125.
13. Botha A. Yeasts in soil. Biodiversity and Ecophysiology of Yeasts: Springer; 2006. p. 221–240.
14. Botha A. The importance and ecology of yeasts in soil. Soil Biol Biochem. 2011; 43: 1–8.
15. Gomes NCM, Fagbola O, Costa R, Rumjanek NG, Buchner A, Mendona-Hagler L, et al. Dynamics of fungal communities in bulk and maize rhizosphere soil in the tropics. Appl Environ Microbiol. 2003; 69: 3758–3766. PMID: 12839741

16. Xu L, Ravnskov S, Larsen J, Nicolaisen M. Linking fungal communities in roots, rhizosphere, and soil to the health status of *Pisum sativum*. FEMS Microbiol Ecol. 2012; 82: 736–745. doi: 10.1111/j.1574-6941.2012.01445.x PMID: 22775574

17. Bahram M, Peay KG, Tedersoo L. Local-scale biogeography and spatiotemporal variability in communities of mycorrhizal fungi. New Phytol. 2015; 205:1454–1463. PMID: 25767850

18. Hartmann M, Lee S, Hallam SJ, Mohn WW. Bacterial, archaeal and eukaryal community structures throughout soil horizons of harvested and naturally disturbed forest stands. Environ Microbiol. 2009; 11:3045–3062. doi: 10.1111/j.1462-2920.2009.02008.x PMID: 19659501

19. Jumpponen A, Jones KL, Blair J. Vertical distribution of fungal communities in tallgrass prairie soil. Mycologia. 2010; 102: 1027–1041. doi: 10.3852/09-316 PMID: 20943503

20. Rime T, Hartmann M, Brunner I, Widmer F, Zeyer J, Frey B. Vertical distribution of the soil microbiota along a successional gradient in a glacier forefield. Mol Ecol. 2015: 24, 1091–1108. doi: 10.1111/mec.13051 PMID: 25533315

21. Eilers KG, Debenport S, Anderson S, Fierer N. Digging deeper to find unique microbial communities: The strong effect of depth on the structure of bacterial and archaeal communities in soil. Soil Biol Biochem. 2012; 50: 58–65.

22. Moll J, Goldmann K, Kramer S, Hempel S, Kandeler E, Marhan S, et al. Resource type and availability regulate fungal communities along arable soil profiles. Microb Ecol. 2015: 1–10.

23. IUSS WGW. World reference base for soil resources 2006, first update 2007. World Soil Resources Reports No. 103. FAO Rome, Italy; 2007.

24. Pausch J, Kuzyakov Y. Soil organic carbon decomposition from recently added and older sources estimated by delta C^{-13} values of CO_2 and organic matter. Soil Biol Biochem. 2012; 55: 40–47.

25. Scharroba A, Dibbern D, Hünninghaus M, Kramer S, Moll J, Butenschoen O, et al. Effects of resource availability and quality on the structure of the micro-food web of an arable soil across depth. Soil Biol Biochem. 2012; 50: 1–11.

26. Renker C, Weisshuhn K, Kellner H, Buscot F. Rationalizing molecular analysis of field-collected roots for assessing diversity of arbuscular mycorrhizal fungi: to pool, or not to pool, that is the question. Mycorrhiza. 2006; 16: 525–531. PMID: 16983569

27. Cocolin L, Bisson L, Mills D. Direct profiling of the yeast dynamics in wine fermentations. FEMS Microbiol Lett. 2000; 189: 81–87. PMID: 10913870

28. Gardes M, Bruns TD. ITS primers with enhanced specificity for basidiomycetes-application to the identification of mycorrhizae and rusts. Mol Ecol. 1993; 2: 113–118. PMID: 8180733

29. Wubet T, Weiss M, Kottke I, Teketay D, Oberwinkler F. Phylogenetic analysis of nuclear small subunit rDNA sequences suggests that the endangered African Pencil Cedar, *Juniperus procera*, is associated with distinct members of *Glomeraceae*. Mycol Res. 2006; 110: 1059–1069. PMID: 16904879

30. Simon L, Lalonde M, Bruns T. Specific amplification of 18S fungal ribosomal genes from vesicular-arbuscular endomycorrhizal fungi colonizing roots. Appl Environ Microbiol. 1992; 58: 291–295. PMID: 1339260

31. Morris EK, Buscot F, Herbst C, Meiners T, Obermaier E, Wäschke NW, et al. Land use and host neighbor identity effects on arbuscular mycorrhizal fungal community composition in focal plant rhizosphere. Biodivers Conserv. 2013; 22: 2193–2205.

32. Hammer Ø, Harper D, Ryan P. PAST: Paleontological statistics software package for education and data analysis. Palaeontol Electron. 2001; 4: 1–9.

33. R Development Core Team. R: A language and environment for statistical computing. R foundation for statistical computing. Vienna, Austria: ISBN 3-900051-07-0, 2013. Available: https://cran.r-project.org/.

34. Anderson MJ. A new method for non-parametric multivariate analysis of variance. Austral Ecol. 2001; 26: 32–46.

35. Oksanen J, Blanchet FG, Kindt R, Legendre P, Minchin P, O'Hara R, et al. vegan: Community Ecology Package. R package version 2.0–2. 2011. Available: https://cran.r-project.org/web/packages/vegan/.

36. Allison SD, Treseder KK. Warming and drying suppress microbial activity and carbon cycling in boreal forest soils. Glob Chang Biol. 2008; 14: 2898–2909.

37. Wubet T, Christ S, Schoning I, Boch S, Gawlich M, Schnabel B, et al. Differences in soil fungal communities between european beech (Fagus sylvatica L.) dominated forests are related to soil and understory vegetation. PLoS ONE. 2012; 7: e47500. doi: 10.1371/journal.pone.0047500 PMID: 23094057

38. Buee M, Reich M, Murat C, Morin E, Nilsson RH, Uroz S, et al. 454 Pyrosequencing analyses of forest soils reveal an unexpectedly high fungal diversity. New Phytol. 2009; 184: 449–456. doi: 10.1111/j.1469-8137.2009.03003.x PMID: 19703112

39. Klaubauf S, Inselsbacher E, Zechmeister-Boltenstern S, Wanek W, Gottsberger R, Strauss J, et al. Molecular diversity of fungal communities in agricultural soils from Lower Austria. Fungal Divers. 2010; 44: 65–75. PMID: 23794962
40. Ma AZ, Zhuang XL, Wu JM, Cui MM, Lv D, Liu CZ, et al. Ascomycota members dominate fungal communities during straw residue decomposition in Arable Soil. PLoS ONE. 2013; 8(6): e66146. doi: 10.1371/journal.pone.0066146 PMID: 23840414
41. de Castro AP, Quirino BF, Pappas G Jr, Kurokawa AS, Neto EL, Kruger RH. Diversity of soil fungal communities of Cerrado and its closely surrounding agriculture fields. Arch Microbiol. 2008; 190: 129–139. doi: 10.1007/s00203-008-0374-6 PMID: 18458875
42. Ciccolini V, Bonari E, Pellegrino E. Land-use intensity and soil properties shape the composition of fungal communities in Mediterranean peaty soils drained for agricultural purposes Biol Fertil Soils. 2015; 51: 719–731.
43. Lauber CL, Strickland MS, Bradford MA, Fierer N. The influence of soil properties on the structure of bacterial and fungal communities across land-use types. Soil Biol Biochem. 2008; 40: 2407–2415.
44. Singh BK, Dawson LA, Macdonald CA, Buckland SM. Impact of biotic and abiotic interaction on soil microbial communities and functions: A field study. Appl Soil Ecol. 2009; 41: 239–248.
45. Waldrop MP, Zak DR, Blackwood CB, Curtis CD, Tilman D. Resource availability controls fungal diversity across a plant diversity gradient. Ecol Lett. 2006; 9: 1127–1135. PMID: 16972876
46. Rousk J, Baath E, Brookes PC, Lauber CL, Lozupone C, Caporaso JG, et al. Soil bacterial and fungal communities across a pH gradient in an arable soil. ISME J. 2010; 4:1340–1351. doi: 10.1038/ismej.2010.58 PMID: 20445636
47. Blaalid R, Carlsen T, Kumar S, Halvorsen R, Ugland KI, Fontana G, et al. Changes in the root-associated fungal communities along a primary succession gradient analysed by 454 pyrosequencing. Mol Ecol. 2012; 21: 1897–1908. PMID: 22590726
48. Bridge PD, Newsham KK. Soil fungal community composition at Mars Oasis, a southern maritime Antarctic site, assessed by PCR amplification and cloning. Fungal Ecol. 2009; 2: 66–74.
49. Selosse MA, Vohnik M, Chauvet E. Out of the rivers: are some aquatic hyphomycetes plant endophytes? New Phytol. 2008; 178: 3–7. doi: 10.1111/j.1469-8137.2008.02390.x PMID: 18315696
50. Kuhnert R, Oberkofler I, Peintner U. Fungal growth and biomass development is boosted by plants in snow-covered soil. Microb Ecol. 2012; 64(1): 79–90. doi: 10.1007/s00248-011-0001-y PMID: 22234510
51. Stursova M, Zifcakova L, Leigh MB, Burgess R, Baldrian P. Cellulose utilization in forest litter and soil: identification of bacterial and fungal decomposers. FEMS Microbiol Ecol. 2012; 80: 735–746. doi: 10.1111/j.1574-6941.2012.01343.x PMID: 22379979
52. Vishniac HS. A multivariate analysis of soil yeasts isolated from a latitudinal gradient. Microb Ecol. 2006; 52: 90–103. PMID: 16708262
53. Connell L, Redman R, Craig S, Rodriguez R. Distribution and abundance of fungi in the soils of Taylor Valley, Antarctica. Soil Biol Biochem. 2006; 38(10):3083–3094.
54. Slavikova E, Vadkertiova R. The diversity of yeasts in the agricultural soil. J Basic Microb. 2003; 43: 430–6.
55. Yurkov AM, Kemler M, Begerow D. Assessment of yeast diversity in soils under different management regimes. Fungal Ecol. 2012; 5: 24–35.
56. Yurkov AM, Kemler M, Begerow D. Species accumulation curves and incidence-based species richness estimators to appraise the diversity of cultivable yeasts from beech forest soils. PLoS ONE. 2011; 6(8). doi: 10.1371/journal.pone.0023671
57. Drigo B, Pijl AS, Duyts H, Kielak A, Gamper HA, Houtekamer MJ, et al. Shifting carbon flow from roots into associated microbial communities in response to elevated atmospheric CO2. PNAS. 2010; 107: 10938–10942. doi: 10.1073/pnas.0912421107 PMID: 20534474
58. Hoffland E, Kuyper TW, Wallander H, Plassard C, Gorbushina AA, Haselwandter K, et al. The role of fungi in weathering. Front Ecol Environ. 2004; 2: 258–264.
59. Money NP. Biomechanics of Invasive Hyphal Growth. In: Howard R, Gow NR, editors. Biology of the Fungal Cell: Springer Berlin Heidelberg; 2007. p. 237–249.
60. Lynch MD, Thorn RG. Diversity of basidiomycetes in michigan agricultural soils. Appl Environ Microbiol. 2006; 72: 7050–7056. PMID: 16950900
61. Cloete KJ, Valentine AJ, Stander MA, Blomerus LM, Botha A. Evidence of symbiosis between the soil yeast Cryptococcus laurentii and a sclerophyllous medicinal shrub, Agathosma betulina (Berg.) Pillans. Microb Ecol. 2009; 57: 624–632. doi: 10.1007/s00248-008-9457-9 PMID: 18958514

62. El-Mehalawy AA, Hassanein NM, Khater HM, El-Din EK, Youssef YA. Influence of maize root colonization by the rhizosphere actinomycetes and yeast fungi on plant growth and on the biological control of late wilt disease. Int J Agric Biol. 2004; 6: 599–605.
63. Renker C, Blanke V, Börstler B, Heinrichs J, Buscot F. Diversity of Cryptococcus and Dioszegia yeasts (Basidiomycota) inhabiting arbuscular mycorrhizal roots or spores. FEMS Yeast Res. 2004; 4: 597–603. PMID: 15040947
64. Becklin KM, Hertweck KL, Jumpponen A. Host identity impacts rhizosphere fungal communities associated with three alpine plant species. Microb Ecol. 2012; 63: 682–693. doi: 10.1007/s00248-011-9968-7 PMID: 22038036
65. König S, Wubet T, Dormann CF, Hempel S, Renker C, Buscot F. TaqMan real-time PCR assays to assess arbuscular mycorrhizal responses to field manipulation of grassland biodiversity: effects of soil characteristics, plant species richness, and functional traits. Appl Environ Microbiol. 2010; 76: 3765–3775. doi: 10.1128/AEM.02951-09 PMID: 20418424
66. Torrecillas E, Alguacil M, Roldán A. Differences in the AMF diversity in soil and roots between two annual and perennial gramineous plants co-occurring in a Mediterranean, semiarid degraded area. Plant Soil. 2011; 354: 97–106.
67. Cuenca G, Lovera M. Seasonal variation and distribution at different soil depths of arbuscular mycorrhizal fungi spores in a tropical sclerophyllous shrubland. Botany. 2010; 88: 54–64.
68. Oehl F, Sieverding E, Ineichen K, Ris EA, Boller T, Wiemken A. Community structure of arbuscular mycorrhizal fungi at different soil depths in extensively and intensively managed agroecosystems. New Phytol. 2005; 165: 273–283. PMID: 15720639
69. Muleta D, Assefa F, Nemomissa S, Granhall U. Distribution of arbuscular mycorrhizal fungi spores in soils of smallholder agroforestry and monocultural coffee systems in southwestern Ethiopia. Biol Fertil Soils. 2008; 44: 653–659.
70. Tian H, Drijber RA, Niu XS, Zhang JL, Li XL. Spatio-temporal dynamics of an indigenous arbuscular mycorrhizal fungal community in an intensively managed maize agroecosystem in North China. Appl Soil Ecol. 2011; 47: 141–152.
71. Higo M, Isobe K, Yamaguchi M, Drijber RA, Jeske ES, Ishii R. Diversity and vertical distribution of indigenous arbuscular mycorrhizal fungi under two soybean rotational systems. Biol Fertil Soils. 2013; 49: 1–12.
72. Gai JP, Gao WJ, Liu L, Chen Q, Feng G, Zhang JL, et al. Infectivity and community composition of arbuscular mycorrhizal fungi from different soil depths in intensively managed agricultural ecosystems. J Soil Sediment. 2015; 15: 1200–1211.
73. Opik M, Moora M, Liira J, Zobel M. Composition of root-colonizing arbuscular mycorrhizal fungal communities in different ecosystems around the globe. J Ecol. 2006; 94: 778–790.
74. Chagnon P-L, Bradley RL, Maherali H, Klironomos JN. A trait-based framework to understand life history of mycorrhizal fungi. Trends Plant Sci. 2013; 18: 484–491. doi: 10.1016/j.tplants.2013.05.001 PMID: 23756036
75. Pausch J, Tian J, Riederer M, Kuzyakov Y. Estimation of rhizodeposition at field scale: upscaling of a 14 C labeling study. Plant Soil. 2013; 364: 273–285.
76. Alguacil MM, Lumini E, Roldan A, Salinas-Garcia JR, Bonfante P, Bianciotto V. The impact of tillage practices on arbuscular mycorrhizal fungal diversity in subtropical crops. Ecol Appl. 2008; 18: 527–536. PMID: 18488613
77. Helgason BL, Walley FL, Germida JJ. No-till soil management increases microbial biomass and alters community profiles in soil aggregates. Appl Soil Ecol. 2010; 46: 390–397.
